# 

**Published:** 1895-04-20

**Authors:** 


### CONTENTS.

**Pay Hospitals or Medical Hotels** - 37

**Desquamation After Scarlet Fever** - 38

**The Causation of Aneurisms.** II.—By A. Pearce Gould, F.R.C.S. - 39

**Modern Sociology.**—Taxes and Tariffs - 40

**Annotations.**—Does Novel Reading Prevent Marriage?—"One Step" at the Tottenham Hospital—Weighing the Baby - 41

**Notes and News** - 42

**The Book World of Medicine and Science** - 53

**Medical Progress and Hospital Clinics**—Club Foot.—III. - 43

Progress in Medicine.—Kidney Diseases - 44

Diseases of the Lungs and Pleuræ - 45

Progress in Surgery.—Genito-Urinary Surgery - 46

**The Institutional Workshop:**

Hospital Administration—An Old-fashioned Election—How not to Elect a House-Surgeon - 49

Practical Departments.—Heating and Ventilation (*continued*) - 51

Hospital Festivals and Meetings - 52

Practical Points - 55

### EXTRA SUPPLEMENT.—THE NURSING MIRROR.

**News from the Nursing World.**—East and West—Princess Louise—A Cancer Hospital—Unauthorised Collections—Women and Sanitation—Difficulties Lightly Met—Ladies at Chelmsford—A Short-sighted Policy—Nurses at Leicester—Queen's Nurses in Wales—A French Hospital School—Homes at Alexandria—Bellevue Training School, U.S.A.—Medical Aid for Women of India—Short Items - xvii
**Elementary Anatomy and Surgery for Nurses.** By W. McAdam Eccles, M.B., M.S., F.R.C.S. - xix
**Appointments** - xix
**Nursing in America—The Convention of Superintendents and Members of the Association** - xx
**Nursing in Germany** - xxi
**Notes from Victoria** - xxi
**Minor Appointments** - xxi
**Magazines for the Month** - xxii
**Novelties for Nurses** - xxii
**For Reading to the Sick** - xxi
**Where to Go** - xxii

